# *rac*-18-Meth­oxy­coronaridine hydro­chloride

**DOI:** 10.1107/S1600536812009981

**Published:** 2012-03-10

**Authors:** Martin E. Kuehne, Rory Waterman

**Affiliations:** a Department of Chemistry, University of Vermont, 82 University Place, Burlington, VT 05405, USA

## Abstract

In the crystal structure of the racemic title compound, C_22_H_29_N_2_O_3_^+^·Cl^−^, both NH groups form N—H⋯Cl hydrogen bonds with the chloride counter-ion, forming translational chains along the *a* axis.

## Related literature

The title compound is a prospective anti-addictive and anti­leishmaniasis agent. For the synsthesis of the free base along with some bio-activity studies, see: Bandarage *et al.* (1995 ▶). For a study of related systems, see: Kuehne *et al.* (2003 ▶).
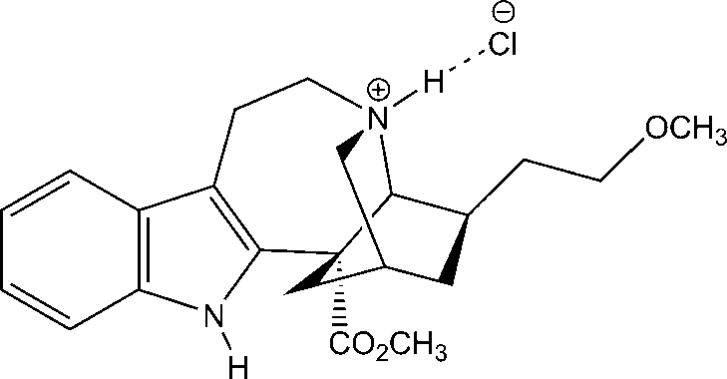


## Experimental

### 

#### Crystal data

C_22_H_29_N_2_O_3_^+^·Cl^−^
*M* *_r_* = 404.92
Monoclinic, 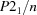

*a* = 9.3692 (14) Å
*b* = 20.161 (3) Å
*c* = 11.6281 (18) Å
β = 110.221 (2)°
*V* = 2061.1 (5) Å^3^
*Z* = 4
Mo *K*α radiation
μ = 0.21 mm^−1^
*T* = 125 K
0.21 × 0.17 × 0.08 mm

#### Data collection

Bruker APEXII CCD diffractometer
Absorption correction: multi-scan (*SADABS*; Bruker, 2007 ▶) *T*_min_ = 0.958, *T*_max_ = 0.983
22802 measured reflections
4374 independent reflections
2620 reflections with *I* > 2σ(*I*)
*R*_int_ = 0.100

#### Refinement

*R*[*F* ^2^ > 2σ(*F* ^2^)] = 0.053
*wR*(*F* ^2^) = 0.118
*S* = 1.01
4374 reflections
261 parameters
H atoms treated by a mixture of independent and constrained refinement
Δρ_max_ = 0.31 e Å^−3^
Δρ_min_ = −0.32 e Å^−3^

### 

Data collection: *APEX2* (Bruker, 2007 ▶); cell refinement: *SAINT* (Bruker, 2007 ▶); data reduction: *SAINT*; program(s) used to solve structure: *SHELXS97* (Sheldrick, 2008 ▶); program(s) used to refine structure: *SHELXL97* (Sheldrick, 2008 ▶); molecular graphics: *SHELXTL* (Sheldrick, 2008 ▶); software used to prepare material for publication: *SHELXTL*.

## Supplementary Material

Crystal structure: contains datablock(s) I, global. DOI: 10.1107/S1600536812009981/ld2049sup1.cif

Supplementary material file. DOI: 10.1107/S1600536812009981/ld2049Isup2.cdx

Structure factors: contains datablock(s) I. DOI: 10.1107/S1600536812009981/ld2049Isup3.hkl

Additional supplementary materials:  crystallographic information; 3D view; checkCIF report

## Figures and Tables

**Table 1:** Hydrogen-bond geometry (Å, °)

*D*—H⋯*A*	*D*—H	H⋯*A*	*D*⋯*A*	*D*—H⋯*A*
N4—H4⋯Cl	1.01 (3)	2.09 (3)	3.054 (2)	160 (3)
N1—H1⋯Cl^i^	0.93 (3)	2.25 (3)	3.157 (2)	165 (2)

Symmetry code: (i) 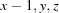.

**Fig. 1.**
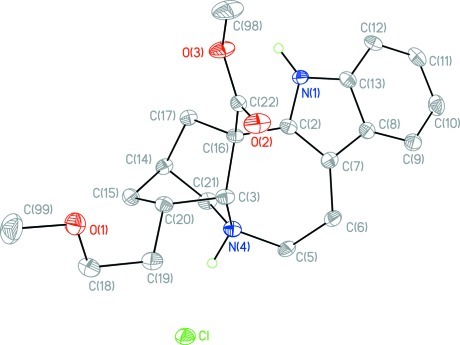
Structure of 18-methoxycoronaridine hydrochloride with elipsoids drawn at the 50% probability level. Hydrogen atoms are omitted for clarity except for H1 and H4.

## supplementary crystallographic information

### Comment

18-Methoxycoronaridine hydrochloride is a compound under investigation for anti-addictive and anti-leishmaniasis activity (Bandarage, *et al.*, 1995) that has prompted a further study on additional, related systems (Kuehne, *et al.*, 2003). Its structure is presented herein to aid these studies. The hydrochloride salt is readily obtained from pure 18-methoxycoronaridine by reaction with anhydrous hydrochloric acid. Though this salt readily crystalizes from a variety of solvents, it was found that ethanol/THF was the optimal system for affording X-ray quality samples. Desipte the conditions, no evidence for decarbomethoxylation was observed.

### Experimental

18-Methoxycoronaridine was prepared according to the literature procedure (Bandarage *et al.*, 1995) and treated in dichloromethane solution with excess of anhydrous HCl. The solvent was removed under reduced pressure, and the the residue was washed with benzene that gave the pure 18-methoxycoronaridine hydrochloride (sub: 195–197 C). X-ray quality crystals were grown from a methanol/THF solvent mixutre.

### Refinement

All non-hydrogen atoms were refined anisotropically. Hydrogens on carbon atoms were placed in calculated positions and refined using a riding model at C—H = 0.95, 0.98 or 1.00 Å and *U*_iso_(H) = 1.2, 1.5 or 1.2 τimes *U*_eq_(C) of the aryl, methyl and methine C-atoms, respectively. Methyl hydrogen atoms were set in staggered orientation and not optimized rotationally.

### Crystal data

**Table:**

C_22_H_29_N_2_O_3_^+^·Cl^−^	*F*(000) = 864
*M**_r_* = 404.92	*D*_x_ = 1.305 Mg m^−^^3^
Monoclinic, *P*2_1_/*n*	Mo *K*α radiation, λ = 0.71073 Å
Hall symbol: -P 2yn	Cell parameters from 4279 reflections
*a* = 9.3692 (14) Å	θ = 2.5–26.9°
*b* = 20.161 (3) Å	µ = 0.21 mm^−^^1^
*c* = 11.6281 (18) Å	*T* = 125 K
β = 110.221 (2)°	Plate, colourless
*V* = 2061.1 (5) Å^3^	0.21 × 0.17 × 0.08 mm
*Z* = 4	

### Data collection

**Table:**

Bruker APEXII CCD diffractometer	4374 independent reflections
Radiation source: fine-focus sealed tube	2620 reflections with *I* > 2σ(*I*)
Graphite monochromator	*R*_int_ = 0.100
φ and ω scans	θ_max_ = 26.7°, θ_min_ = 2.0°
Absorption correction: multi-scan (*SADABS*; Bruker, 2007)	*h* = −11→11
*T*_min_ = 0.958, *T*_max_ = 0.983	*k* = −25→25
22802 measured reflections	*l* = −14→14

### Refinement

**Table:**

Refinement on *F*^2^	Primary atom site location: structure-invariant direct methods
Least-squares matrix: full	Secondary atom site location: difference Fourier map
*R*[*F*^2^ > 2σ(*F*^2^)] = 0.053	Hydrogen site location: inferred from neighbouring sites
*wR*(*F*^2^) = 0.118	H atoms treated by a mixture of independent and constrained refinement
*S* = 1.01	*w* = 1/[σ^2^(*F*_o_^2^) + (0.0445*P*)^2^ + 0.8261*P*] where *P* = (*F*_o_^2^ + 2*F*_c_^2^)/3
4374 reflections	(Δ/σ)_max_ < 0.001
261 parameters	Δρ_max_ = 0.31 e Å^−^^3^
0 restraints	Δρ_min_ = −0.32 e Å^−^^3^

### Special details

**Table:**

Geometry. All e.s.d.'s (except the e.s.d. in the dihedral angle between two l.s. planes) are estimated using the full covariance matrix. The cell e.s.d.'s are taken into account individually in the estimation of e.s.d.'s in distances, angles and torsion angles; correlations between e.s.d.'s in cell parameters are only used when they are defined by crystal symmetry. An approximate (isotropic) treatment of cell e.s.d.'s is used for estimating e.s.d.'s involving l.s. planes.
Refinement. Suitable crystals were mounted in a nylon loop with Paratone-*N* cryoprotectant oil and data collected on a Bruker *APEX* 2 CCD platform diffractometer. The structure was solved using direct methods and standard difference map techniques, and was refined by full-matrix least-squares procedures on F2 with *SHELXTL* Version 6.14 (Sheldrick, 2008).Refinement of *F*^2^ against ALL reflections. The weighted *R*-factor *wR* and goodness of fit *S* are based on *F*^2^, conventional *R*-factors *R* are based on *F*, with *F* set to zero for negative *F*^2^. The threshold expression of *F*^2^ > σ(*F*^2^) is used only for calculating *R*-factors(gt) *etc*. and is not relevant to the choice of reflections for refinement. *R*-factors based on *F*^2^ are statistically about twice as large as those based on *F*, and *R*- factors based on ALL data will be even larger.

### Fractional atomic coordinates and isotropic or equivalent isotropic displacement parameters (Å^2^)

**Table:**

	*x*	*y*	*z*	*U*_iso_*/*U*_eq_	
C2	−0.6298 (3)	−0.03049 (13)	0.2437 (2)	0.0159 (6)	
C3	−0.3327 (3)	0.04266 (13)	0.4114 (2)	0.0204 (6)	
H11A	−0.2318	0.0509	0.4745	0.024*	
H11B	−0.3874	0.0099	0.4441	0.024*	
C5	−0.2795 (3)	−0.05676 (13)	0.2987 (2)	0.0195 (6)	
H10A	−0.1936	−0.0632	0.2687	0.023*	
H10B	−0.2456	−0.0720	0.3849	0.023*	
C6	−0.4102 (3)	−0.10020 (13)	0.2237 (2)	0.0194 (6)	
H12C	−0.4299	−0.0918	0.1357	0.023*	
H12D	−0.3796	−0.1472	0.2402	0.023*	
C7	−0.5551 (3)	−0.08957 (13)	0.2490 (2)	0.0178 (6)	
C8	−0.6388 (3)	−0.13907 (13)	0.2870 (2)	0.0177 (6)	
C9	−0.6179 (3)	−0.20695 (13)	0.3122 (2)	0.0224 (6)	
H12A	−0.5347	−0.2298	0.3015	0.027*	
C10	−0.7204 (3)	−0.24016 (14)	0.3529 (3)	0.0268 (7)	
H12B	−0.7063	−0.2861	0.3714	0.032*	
C11	−0.8452 (3)	−0.20713 (14)	0.3674 (2)	0.0249 (7)	
H11E	−0.9144	−0.2313	0.3949	0.030*	
C12	−0.8691 (3)	−0.14076 (13)	0.3427 (2)	0.0204 (6)	
H18A	−0.9534	−0.1186	0.3528	0.024*	
C13	−0.7655 (3)	−0.10689 (13)	0.3021 (2)	0.0179 (6)	
C14	−0.4234 (3)	0.10731 (13)	0.3792 (2)	0.0180 (6)	
H10C	−0.4145	0.1323	0.4557	0.022*	
C15	−0.3613 (3)	0.14888 (13)	0.2976 (2)	0.0198 (6)	
H19A	−0.2491	0.1518	0.3343	0.024*	
H19B	−0.4034	0.1944	0.2898	0.024*	
C16	−0.5960 (3)	0.03842 (12)	0.2080 (2)	0.0152 (6)	
C17	−0.5909 (3)	0.09052 (13)	0.3084 (2)	0.0178 (6)	
H11C	−0.6468	0.1310	0.2699	0.021*	
H11D	−0.6393	0.0722	0.3649	0.021*	
C18	−0.2695 (3)	0.19660 (14)	0.0775 (3)	0.0237 (6)	
H11H	−0.2384	0.2229	0.1541	0.028*	
H11I	−0.1882	0.2000	0.0415	0.028*	
C19	−0.2917 (3)	0.12482 (13)	0.1052 (2)	0.0212 (6)	
H11F	−0.1927	0.1062	0.1575	0.025*	
H11G	−0.3258	0.0995	0.0275	0.025*	
C20	−0.4076 (3)	0.11564 (12)	0.1697 (2)	0.0170 (6)	
H18B	−0.5047	0.1368	0.1170	0.020*	
C21	−0.4422 (3)	0.04253 (13)	0.1856 (2)	0.0172 (6)	
H17A	−0.4478	0.0167	0.1108	0.021*	
C22	−0.7202 (3)	0.05207 (13)	0.0837 (2)	0.0165 (6)	
C98	−0.9699 (3)	0.08902 (16)	−0.0188 (3)	0.0345 (8)	
H12E	−1.0560	0.1086	−0.0015	0.052*	
H12F	−1.0004	0.0462	−0.0600	0.052*	
H12G	−0.9381	0.1189	−0.0719	0.052*	
C99	−0.3917 (4)	0.28863 (14)	−0.0384 (3)	0.0377 (8)	
H11J	−0.4889	0.3044	−0.0963	0.057*	
H11K	−0.3136	0.2912	−0.0766	0.057*	
H11L	−0.3617	0.3164	0.0354	0.057*	
N1	−0.7580 (2)	−0.04105 (11)	0.27505 (19)	0.0167 (5)	
N4	−0.3142 (2)	0.01673 (11)	0.29607 (19)	0.0174 (5)	
H4	−0.214 (4)	0.0360 (15)	0.299 (3)	0.048 (10)*	
O1	−0.4075 (2)	0.22182 (9)	−0.00591 (17)	0.0262 (5)	
O2	−0.7097 (2)	0.03754 (9)	−0.01348 (16)	0.0256 (5)	
O3	−0.84383 (19)	0.07907 (9)	0.09564 (16)	0.0246 (5)	
Cl	0.02001 (7)	0.05216 (3)	0.35272 (6)	0.02537 (18)	
H1	−0.824 (3)	−0.0090 (14)	0.285 (2)	0.027 (8)*	

### Atomic displacement parameters (Å^2^)

**Table:**

	*U*^11^	*U*^22^	*U*^33^	*U*^12^	*U*^13^	*U*^23^
C2	0.0138 (13)	0.0207 (15)	0.0116 (12)	−0.0009 (11)	0.0022 (10)	0.0002 (11)
C3	0.0189 (14)	0.0267 (16)	0.0138 (13)	0.0020 (12)	0.0034 (11)	−0.0005 (11)
C5	0.0169 (14)	0.0194 (15)	0.0208 (14)	0.0057 (12)	0.0049 (11)	0.0016 (12)
C6	0.0181 (14)	0.0185 (15)	0.0218 (14)	0.0031 (11)	0.0070 (11)	−0.0004 (12)
C7	0.0169 (14)	0.0206 (15)	0.0143 (13)	0.0019 (11)	0.0032 (11)	−0.0017 (11)
C8	0.0166 (14)	0.0199 (15)	0.0132 (13)	−0.0003 (11)	0.0011 (11)	−0.0018 (11)
C9	0.0188 (15)	0.0226 (16)	0.0223 (15)	0.0011 (12)	0.0027 (12)	−0.0009 (12)
C10	0.0295 (17)	0.0168 (15)	0.0300 (17)	−0.0018 (12)	0.0052 (13)	0.0031 (13)
C11	0.0215 (15)	0.0263 (17)	0.0246 (16)	−0.0081 (12)	0.0051 (13)	0.0018 (12)
C12	0.0133 (14)	0.0265 (16)	0.0192 (14)	−0.0028 (11)	0.0030 (11)	0.0002 (12)
C13	0.0152 (14)	0.0218 (15)	0.0135 (13)	−0.0018 (11)	0.0009 (11)	−0.0001 (11)
C14	0.0174 (13)	0.0205 (15)	0.0147 (13)	0.0023 (11)	0.0039 (11)	−0.0026 (11)
C15	0.0162 (14)	0.0202 (15)	0.0209 (14)	−0.0022 (11)	0.0038 (11)	−0.0034 (12)
C16	0.0108 (13)	0.0189 (15)	0.0140 (13)	0.0011 (10)	0.0018 (10)	0.0008 (11)
C17	0.0170 (14)	0.0197 (15)	0.0170 (14)	0.0015 (11)	0.0062 (11)	−0.0001 (11)
C18	0.0190 (15)	0.0273 (16)	0.0254 (15)	−0.0024 (12)	0.0085 (12)	0.0014 (13)
C19	0.0179 (14)	0.0253 (16)	0.0204 (15)	0.0014 (12)	0.0065 (12)	0.0016 (12)
C20	0.0141 (13)	0.0195 (15)	0.0159 (13)	0.0011 (11)	0.0035 (11)	0.0024 (11)
C21	0.0134 (13)	0.0230 (16)	0.0133 (13)	0.0013 (11)	0.0021 (10)	−0.0004 (11)
C22	0.0151 (13)	0.0151 (14)	0.0192 (14)	−0.0010 (11)	0.0059 (11)	0.0007 (11)
C98	0.0188 (16)	0.052 (2)	0.0237 (16)	0.0125 (14)	−0.0035 (13)	−0.0020 (15)
C99	0.051 (2)	0.0228 (18)	0.044 (2)	0.0058 (15)	0.0223 (17)	0.0096 (15)
N1	0.0133 (11)	0.0181 (13)	0.0187 (12)	0.0011 (9)	0.0052 (9)	−0.0012 (9)
N4	0.0141 (12)	0.0219 (13)	0.0135 (11)	0.0006 (9)	0.0013 (9)	0.0005 (9)
O1	0.0247 (11)	0.0212 (11)	0.0329 (12)	0.0015 (8)	0.0103 (9)	0.0061 (9)
O2	0.0212 (10)	0.0398 (13)	0.0148 (10)	0.0043 (9)	0.0049 (8)	−0.0033 (9)
O3	0.0162 (10)	0.0372 (12)	0.0175 (10)	0.0087 (8)	0.0019 (8)	−0.0009 (8)
Cl	0.0161 (3)	0.0283 (4)	0.0299 (4)	0.0018 (3)	0.0056 (3)	−0.0024 (3)

### Geometric parameters (Å, º)

**Table:**

C2—C7	1.372 (4)	C15—H19B	0.9900
C2—N1	1.387 (3)	C16—C22	1.535 (3)
C2—C16	1.514 (3)	C16—C21	1.552 (3)
C3—N4	1.504 (3)	C16—C17	1.559 (3)
C3—C14	1.530 (3)	C17—H11C	0.9900
C3—H11A	0.9900	C17—H11D	0.9900
C3—H11B	0.9900	C18—O1	1.416 (3)
C5—C6	1.513 (3)	C18—C19	1.512 (4)
C5—N4	1.515 (3)	C18—H11H	0.9900
C5—H10A	0.9900	C18—H11I	0.9900
C5—H10B	0.9900	C19—C20	1.530 (3)
C6—C7	1.500 (3)	C19—H11F	0.9900
C6—H12C	0.9900	C19—H11G	0.9900
C6—H12D	0.9900	C20—C21	1.534 (4)
C7—C8	1.430 (4)	C20—H18B	1.0000
C8—C9	1.399 (4)	C21—N4	1.515 (3)
C8—C13	1.416 (4)	C21—H17A	1.0000
C9—C10	1.381 (4)	C22—O2	1.204 (3)
C9—H12A	0.9500	C22—O3	1.330 (3)
C10—C11	1.406 (4)	C98—O3	1.456 (3)
C10—H12B	0.9500	C98—H12E	0.9800
C11—C12	1.371 (4)	C98—H12F	0.9800
C11—H11E	0.9500	C98—H12G	0.9800
C12—C13	1.396 (4)	C99—O1	1.420 (3)
C12—H18A	0.9500	C99—H11J	0.9800
C13—N1	1.372 (3)	C99—H11K	0.9800
C14—C15	1.524 (4)	C99—H11L	0.9800
C14—C17	1.537 (3)	N1—H1	0.93 (3)
C14—H10C	1.0000	N4—Cl	3.054 (2)
C15—C20	1.550 (3)	N4—H4	1.01 (3)
C15—H19A	0.9900		
			
C7—C2—N1	109.2 (2)	C14—C17—C16	108.1 (2)
C7—C2—C16	131.0 (2)	C14—C17—H11C	110.1
N1—C2—C16	119.7 (2)	C16—C17—H11C	110.1
N4—C3—C14	107.7 (2)	C14—C17—H11D	110.1
N4—C3—H11A	110.2	C16—C17—H11D	110.1
C14—C3—H11A	110.2	H11C—C17—H11D	108.4
N4—C3—H11B	110.2	O1—C18—C19	109.2 (2)
C14—C3—H11B	110.2	O1—C18—H11H	109.8
H11A—C3—H11B	108.5	C19—C18—H11H	109.8
C6—C5—N4	115.4 (2)	O1—C18—H11I	109.8
C6—C5—H10A	108.4	C19—C18—H11I	109.8
N4—C5—H10A	108.4	H11H—C18—H11I	108.3
C6—C5—H10B	108.4	C18—C19—C20	113.2 (2)
N4—C5—H10B	108.4	C18—C19—H11F	108.9
H10A—C5—H10B	107.5	C20—C19—H11F	108.9
C7—C6—C5	114.3 (2)	C18—C19—H11G	108.9
C7—C6—H12C	108.7	C20—C19—H11G	108.9
C5—C6—H12C	108.7	H11F—C19—H11G	107.8
C7—C6—H12D	108.7	C19—C20—C21	113.0 (2)
C5—C6—H12D	108.7	C19—C20—C15	114.1 (2)
H12C—C6—H12D	107.6	C21—C20—C15	107.5 (2)
C2—C7—C8	107.1 (2)	C19—C20—H18B	107.3
C2—C7—C6	126.7 (2)	C21—C20—H18B	107.3
C8—C7—C6	126.2 (2)	C15—C20—H18B	107.3
C9—C8—C13	119.0 (2)	N4—C21—C20	107.1 (2)
C9—C8—C7	134.0 (2)	N4—C21—C16	110.39 (19)
C13—C8—C7	106.9 (2)	C20—C21—C16	108.6 (2)
C10—C9—C8	118.8 (3)	N4—C21—H17A	110.2
C10—C9—H12A	120.6	C20—C21—H17A	110.2
C8—C9—H12A	120.6	C16—C21—H17A	110.2
C9—C10—C11	121.1 (3)	O2—C22—O3	123.7 (2)
C9—C10—H12B	119.4	O2—C22—C16	124.0 (2)
C11—C10—H12B	119.4	O3—C22—C16	112.3 (2)
C12—C11—C10	121.3 (3)	O3—C98—H12E	109.5
C12—C11—H11E	119.3	O3—C98—H12F	109.5
C10—C11—H11E	119.3	H12E—C98—H12F	109.5
C11—C12—C13	117.8 (3)	O3—C98—H12G	109.5
C11—C12—H18A	121.1	H12E—C98—H12G	109.5
C13—C12—H18A	121.1	H12F—C98—H12G	109.5
N1—C13—C12	130.4 (2)	O1—C99—H11J	109.5
N1—C13—C8	107.7 (2)	O1—C99—H11K	109.5
C12—C13—C8	121.9 (2)	H11J—C99—H11K	109.5
C15—C14—C3	108.7 (2)	O1—C99—H11L	109.5
C15—C14—C17	109.0 (2)	H11J—C99—H11L	109.5
C3—C14—C17	108.8 (2)	H11K—C99—H11L	109.5
C15—C14—H10C	110.1	C13—N1—C2	109.0 (2)
C3—C14—H10C	110.1	C13—N1—H1	123.8 (17)
C17—C14—H10C	110.1	C2—N1—H1	126.9 (17)
C14—C15—C20	108.6 (2)	C3—N4—C21	109.59 (19)
C14—C15—H19A	110.0	C3—N4—C5	114.3 (2)
C20—C15—H19A	110.0	C21—N4—C5	117.03 (19)
C14—C15—H19B	110.0	C3—N4—Cl	98.72 (14)
C20—C15—H19B	110.0	C21—N4—Cl	123.72 (15)
H19A—C15—H19B	108.3	C5—N4—Cl	91.58 (13)
C2—C16—C22	105.1 (2)	C3—N4—H4	103.3 (18)
C2—C16—C21	112.9 (2)	C21—N4—H4	110.6 (18)
C22—C16—C21	106.13 (19)	C5—N4—H4	100.7 (18)
C2—C16—C17	111.6 (2)	Cl—N4—H4	13.3 (18)
C22—C16—C17	113.6 (2)	C18—O1—C99	111.6 (2)
C21—C16—C17	107.45 (19)	C22—O3—C98	114.9 (2)
			
N4—C5—C6—C7	−51.3 (3)	C18—C19—C20—C15	−62.8 (3)
N1—C2—C7—C8	−0.9 (3)	C14—C15—C20—C19	−147.6 (2)
C16—C2—C7—C8	−179.2 (2)	C14—C15—C20—C21	−21.4 (3)
N1—C2—C7—C6	−179.1 (2)	C19—C20—C21—N4	80.5 (2)
C16—C2—C7—C6	2.7 (4)	C15—C20—C21—N4	−46.3 (3)
C5—C6—C7—C2	54.5 (4)	C19—C20—C21—C16	−160.3 (2)
C5—C6—C7—C8	−123.3 (3)	C15—C20—C21—C16	72.9 (2)
C2—C7—C8—C9	−177.7 (3)	C2—C16—C21—N4	−54.3 (3)
C6—C7—C8—C9	0.4 (5)	C22—C16—C21—N4	−168.9 (2)
C2—C7—C8—C13	0.7 (3)	C17—C16—C21—N4	69.2 (2)
C6—C7—C8—C13	178.9 (2)	C2—C16—C21—C20	−171.4 (2)
C13—C8—C9—C10	−1.0 (4)	C22—C16—C21—C20	74.0 (2)
C7—C8—C9—C10	177.3 (3)	C17—C16—C21—C20	−47.9 (2)
C8—C9—C10—C11	0.9 (4)	C2—C16—C22—O2	−88.7 (3)
C9—C10—C11—C12	−0.5 (4)	C21—C16—C22—O2	31.2 (3)
C10—C11—C12—C13	0.2 (4)	C17—C16—C22—O2	149.1 (3)
C11—C12—C13—N1	−177.4 (2)	C2—C16—C22—O3	88.4 (2)
C11—C12—C13—C8	−0.3 (4)	C21—C16—C22—O3	−151.7 (2)
C9—C8—C13—N1	178.4 (2)	C17—C16—C22—O3	−33.8 (3)
C7—C8—C13—N1	−0.3 (3)	C12—C13—N1—C2	177.2 (3)
C9—C8—C13—C12	0.8 (4)	C8—C13—N1—C2	−0.2 (3)
C7—C8—C13—C12	−178.0 (2)	C7—C2—N1—C13	0.7 (3)
N4—C3—C14—C15	−44.1 (3)	C16—C2—N1—C13	179.2 (2)
N4—C3—C14—C17	74.5 (2)	C14—C3—N4—C21	−25.3 (3)
C3—C14—C15—C20	71.2 (3)	C14—C3—N4—C5	−158.9 (2)
C17—C14—C15—C20	−47.2 (3)	C14—C3—N4—Cl	105.34 (18)
C7—C2—C16—C22	108.9 (3)	C20—C21—N4—C3	75.1 (2)
N1—C2—C16—C22	−69.2 (3)	C16—C21—N4—C3	−42.9 (3)
C7—C2—C16—C21	−6.4 (4)	C20—C21—N4—C5	−152.6 (2)
N1—C2—C16—C21	175.5 (2)	C16—C21—N4—C5	89.4 (2)
C7—C2—C16—C17	−127.5 (3)	C20—C21—N4—Cl	−40.4 (2)
N1—C2—C16—C17	54.4 (3)	C16—C21—N4—Cl	−158.41 (15)
C15—C14—C17—C16	72.0 (3)	C6—C5—N4—C3	105.4 (2)
C3—C14—C17—C16	−46.4 (3)	C6—C5—N4—C21	−24.7 (3)
C2—C16—C17—C14	103.9 (2)	C6—C5—N4—Cl	−154.30 (19)
C22—C16—C17—C14	−137.5 (2)	C19—C18—O1—C99	−178.0 (2)
C21—C16—C17—C14	−20.4 (3)	O2—C22—O3—C98	1.2 (4)
O1—C18—C19—C20	−63.6 (3)	C16—C22—O3—C98	−175.9 (2)
C18—C19—C20—C21	174.0 (2)		

### Hydrogen-bond geometry (Å, º)

**Table:**

*D*—H···*A*	*D*—H	H···*A*	*D*···*A*	*D*—H···*A*
N4—H4···Cl	1.01 (3)	2.09 (3)	3.054 (2)	160 (3)
N1—H1···Cl^i^	0.93 (3)	2.25 (3)	3.157 (2)	165 (2)

Symmetry code: (i) *x*−1, *y*, *z*.
